# Floating Elbow in Children as a Serious Domestic Accident: A Case Report

**DOI:** 10.7759/cureus.77107

**Published:** 2025-01-07

**Authors:** María Mercedes Medina Villate, Alberto Daniel Navarro Vergara

**Affiliations:** 1 Orthopaedics and Traumatology, Hospital de Especialidades Quirúrgicas Ingavi IPS, Asunción, PRY; 2 Orthopaedics and Traumatology, Hospital de Trauma “Manuel Giagni”, Asunción, PRY

**Keywords:** both bone forearm fracture, covid-19 pandemic, domestic accident, early surgical intervention, floating elbow, intramedullary fixation, metaphyseal-diaphyseal junctional humerus fracture, parental awareness, pediatric orthopedic injury, radial nerve injury

## Abstract

The "floating elbow" is a rare and complex traumatic injury predominantly seen in the pediatric population. It is characterized by simultaneous fractures of the humerus and one or both forearm bones. This article delves into the case of a three-year-old boy who sustained severe injuries to his right arm following a domestic accident involving a centrifuge. The child presented with a Grade IIIA (Gustilo/Anderson classification) open fracture of the humerus, accompanied by fractures in both forearm bones. In response to this critical situation, emergency surgery was performed, initially employing damage control techniques with an external fixator debridement and irrigation. This was followed by a secondary operation utilizing intramedullary titanium elastic nails (TENs) to stabilize the fractures. Despite encountering complications such as radial nerve neuropraxia and wrist drop, the patient demonstrated a promising recovery trajectory. He regained functional elbow movement and did not suffer from any disabling nerve damage such as nervous palsy. This case highlights the increased risk of domestic accidents, particularly during the COVID-19 pandemic, when children spend more time at home, thus becoming more susceptible to such injuries. The article underscores the critical role of surgical intervention in managing complex fractures and advocates for increased parental vigilance to prevent domestic accidents in children.

## Introduction

Accidents rank among the leading causes of mortality worldwide, with domestic accidents holding a significant share of this burden [1]. During the pandemic, children were particularly vulnerable to domestic accidents, likely due to increased time spent at home and parental decisions made under unprecedented circumstances [2]. Research indicates that domestic accidents are most prevalent during the first year of life, affecting 84% of children, and tend to decrease in frequency by the second and third years, affecting 75% and 50% of children, respectively [3].

The term "floating elbow" refers to the simultaneous occurrence of a humeral supracondylar fracture and a fracture of one or both forearm bones [4]. In orthopedic trauma, "floating" describes fractures occurring above and below a joint. This condition is more commonly observed in males and is predominantly caused by motor vehicle accidents, followed by industrial accidents and falls [5]. However, when considering pediatric patients, falls account for most cases, comprising 96% of incidents [6]. Functional outcomes vary, with poorer results associated with open fractures and improved outcomes linked to single-stage surgical interventions [7]. 

Floating elbow injuries are unique and complex in children because they generate wrist and joint dissociation, as well as metaphyseal injuries [8]. 

A severe classification system exists, categorizing injuries into classes: Class 1 includes Gartland 1, Class 2 includes Gartland 2, and Class 3 encompasses Gartland 3 and 4. Class 3 injuries are notably associated with a higher incidence of nerve injury and a rare occurrence of compartment syndrome (0.2%) [9].

In the absence of a standardized classification for floating elbow in children, we are also guided by the one proposed by the Hospital de Traumatología “Victorio de la Fuente Narváez” where, through clinical and radiological assessment, a classification is made from I to VI that indicates the prognosis of how the injury will evolve, with the worst prognosis being VI and the best prognosis being I [10].

Among the most important challenges and determinants of a good prognosis, we can mention the speed of surgical interposition, the right technique, and the correct osteosynthesis materials. 

In this report, we present a case involving ipsilateral fractures of the humerus and forearm bones accompanied by severe soft tissue damage. Our focus is on the traumatic event, the severity of the injuries, and the immediate complications that ensue.

## Case presentation

In this case report, we present the clinical journey of a three-year-old boy who suffered a severe upper limb injury due to a domestic accident involving a centrifuge machine (in our country these machines are used to dry clothes, they have as large metal picks that rotate on an axis to dry the clothes, these machines usually have lids but sometimes no longer have). While this machine is not found in every country in the world, there are others that have the same mechanism and can cause similar injuries. The mechanism of injury was torsional and high-energy, resulting in complex fractures and soft tissue damage, and the following tissues were involved: cutaneous, subcutaneous, muscular, adipose, endothelial, nervous, and osseous.

**Figure 1:**
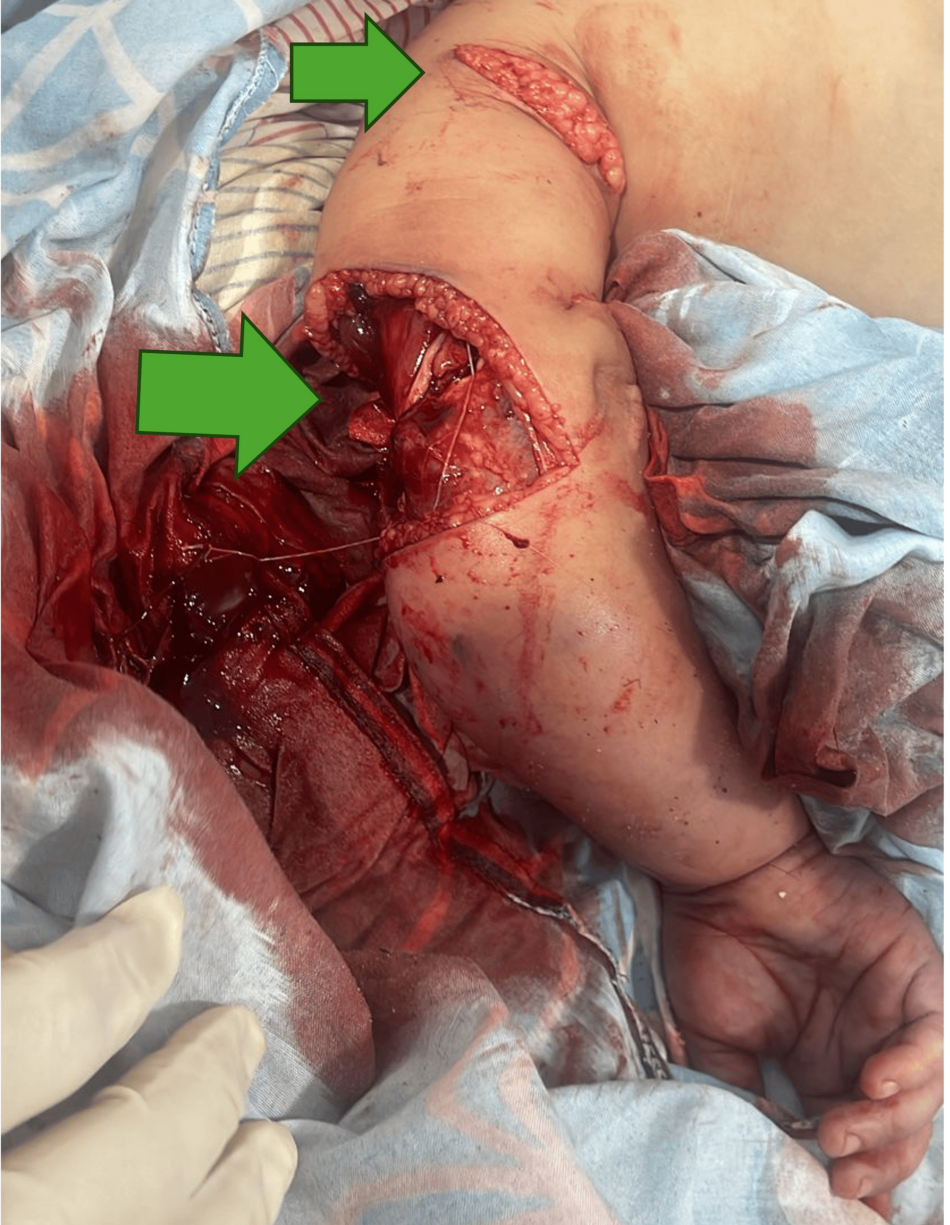
Soft tissue injuries, posterior view The trauma mechanism was torsional and with high-energy, posterior view. Cutaneous, subcutaneous muscular, adipose, endothelial, and endothelial involvement is observed.

**Figure 2:**
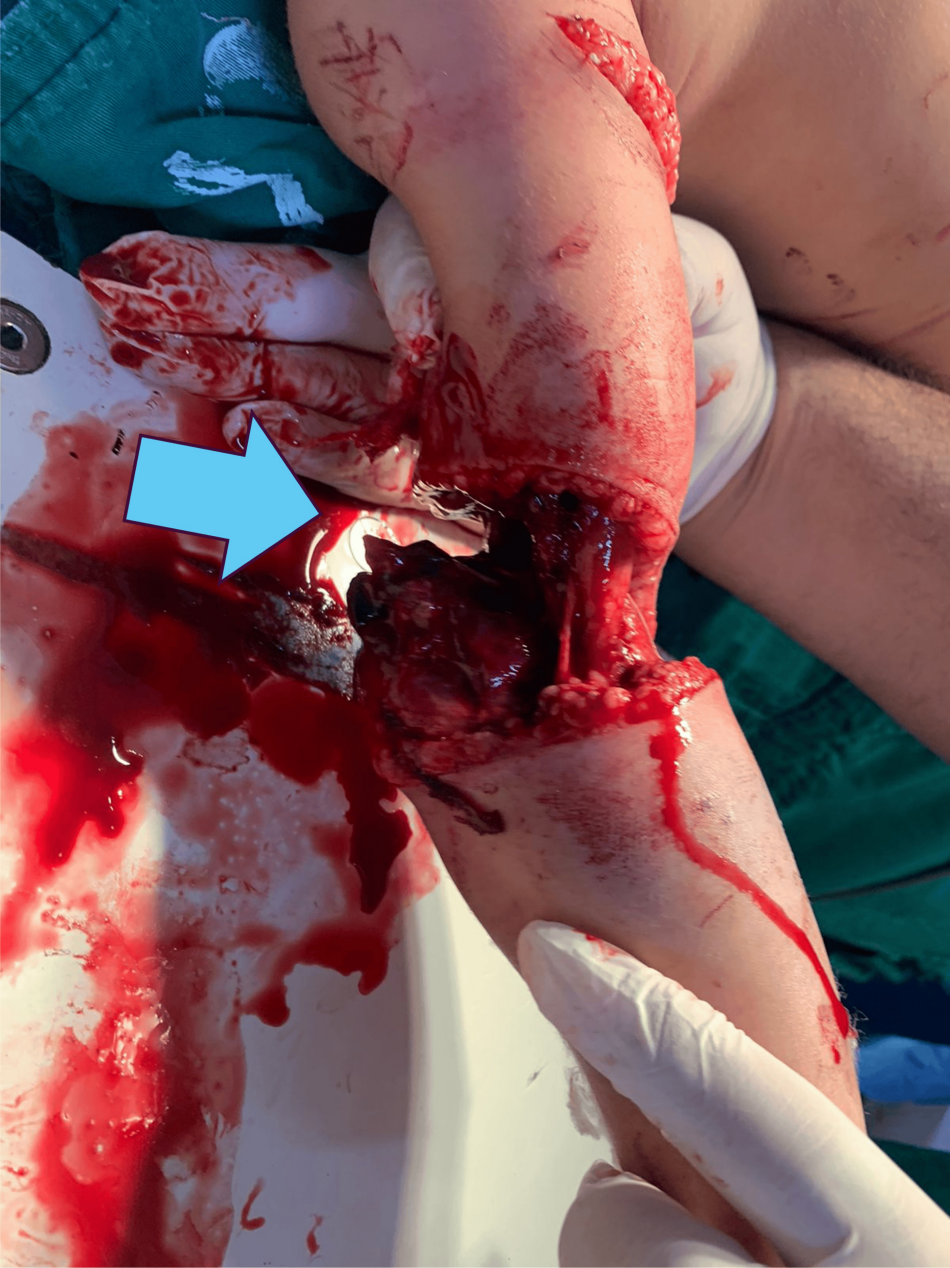
Soft tissue injuries, anterior view The mechanism of high-energy torsional trauma with extensive soft tissue and bone damage is observed. Cutaneous, subcutaneous muscular, adipose, endothelial, and osseous involvement is observed.

The young patient was brought to the emergency department with an open wound on his right forearm. The mother recounted that the child in an oversight had inserted his dominant upper limb into a functioning centrifuge, leading to the injury. Upon general examination, no other significant injuries were noted. However, the specific physical examination revealed a child in distress, with a painful expression and functional impairment of the right upper limb. A large wound was observed at the elbow and proximal third of the inner arm, with bone exposure at the elbow, edematous muscle tissue, and inadequate coverage, thus demonstrating that the trauma mechanism was torsional and high energy (Figures 1-2). Despite the severity of the injury, distal pulses were present, and capillary refill in the fingers was intact. Due to intense pain, distal sensation and motor function could not be assessed.

**Figure 3:**
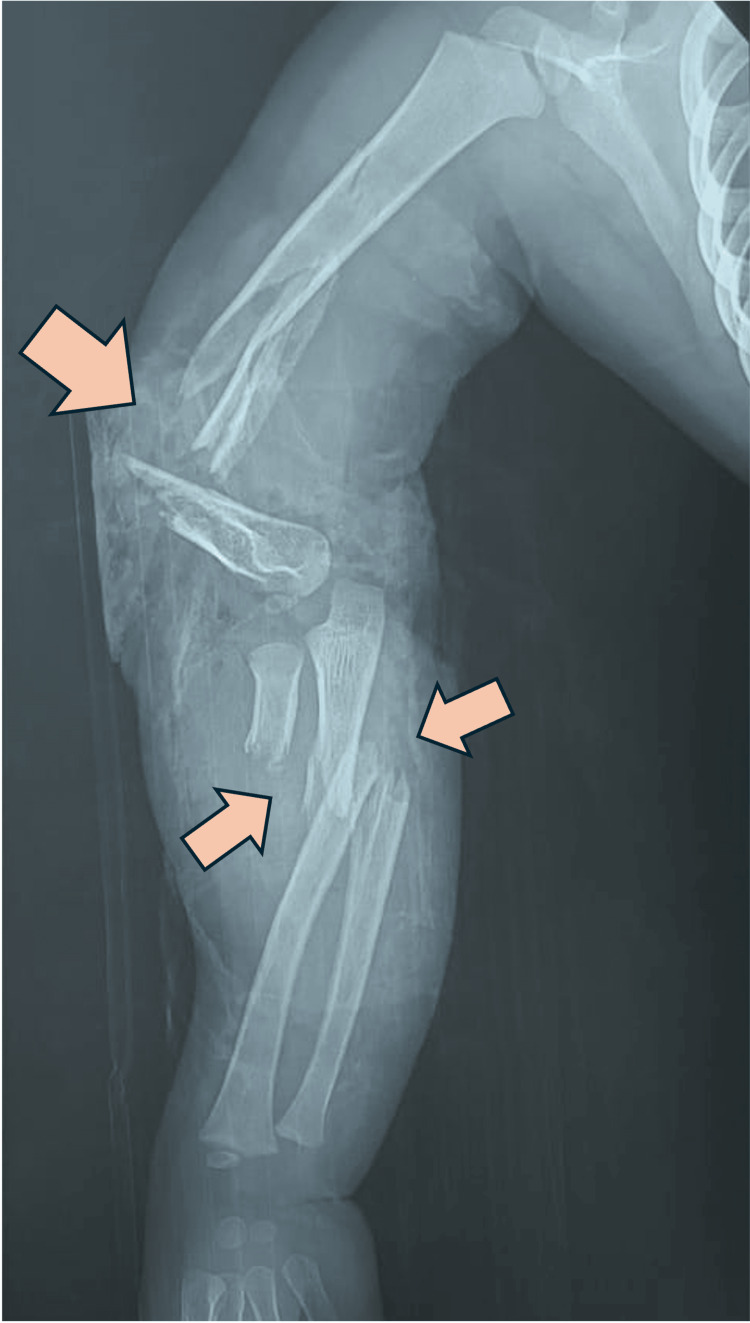
X-ray taken in the emergency room in the anteroposterior (AP) position Diaphyseal fracture of the humerus with a third fragment and a long spiral fracture line was confirmed, with a fracture of both bones of the forearm, featuring a transverse fracture in the radius with significant displacement and a spiral fracture in the ulna.

Auxiliary radiographic studies confirmed a Grade IIIA open diaphyseal fracture of the humerus with a third fragment and a long spiral fracture line. In addition, both bones of the forearm were fractured, with a transverse fracture in the radius showing significant displacement and a spiral fracture in the ulna (Figure 3).

**Figure 4:**
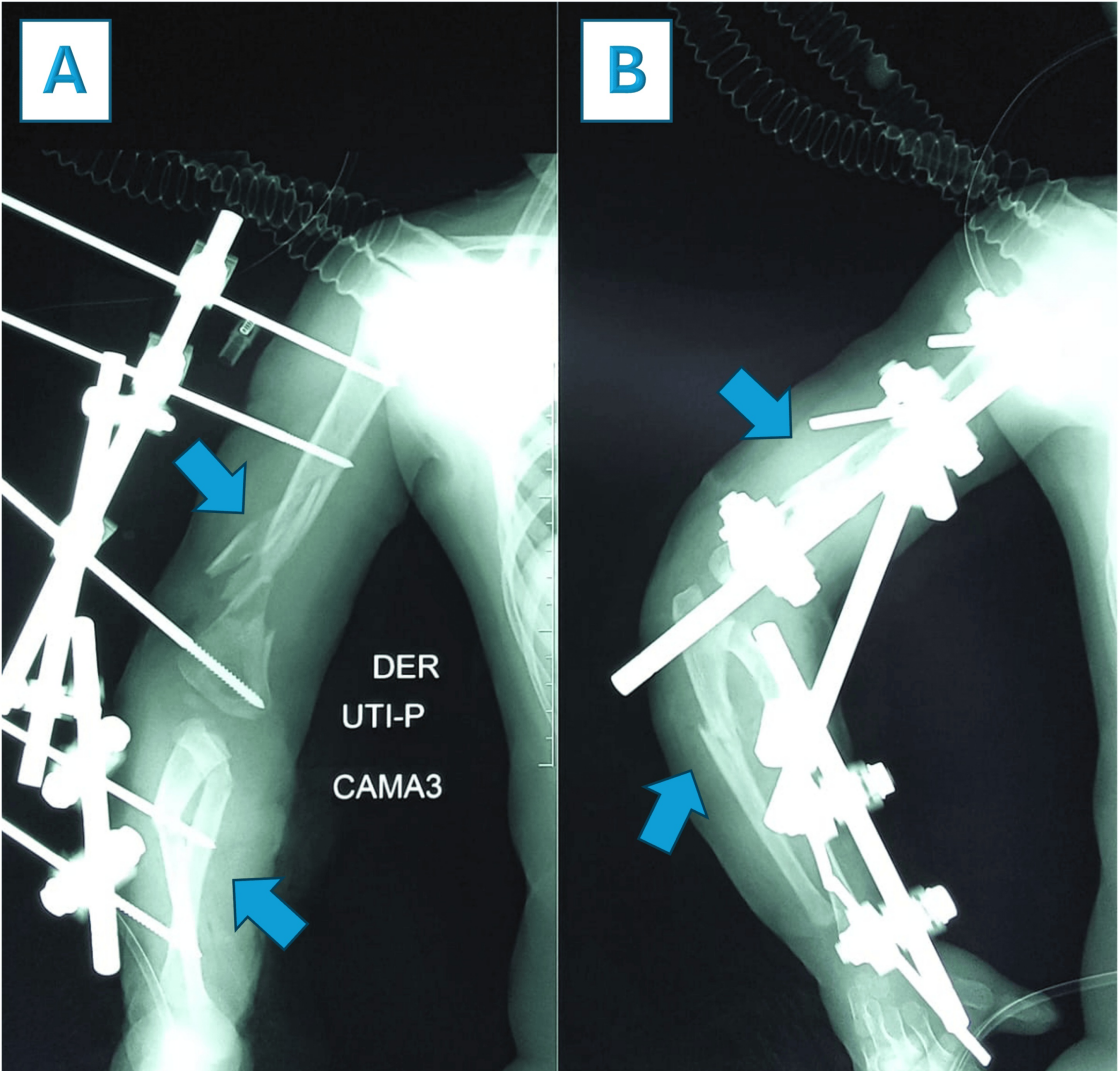
Postoperative X-rays in the anteroposterior (AP) and lateral views after damage control with external fixator stabilization An acceptable reduction of the humeral bone injury and alignment of the forearm fractures was achieved, as shown in the postoperative X-ray in the AP view (Image A) and lateral view (Image B).

**Figure 5:**
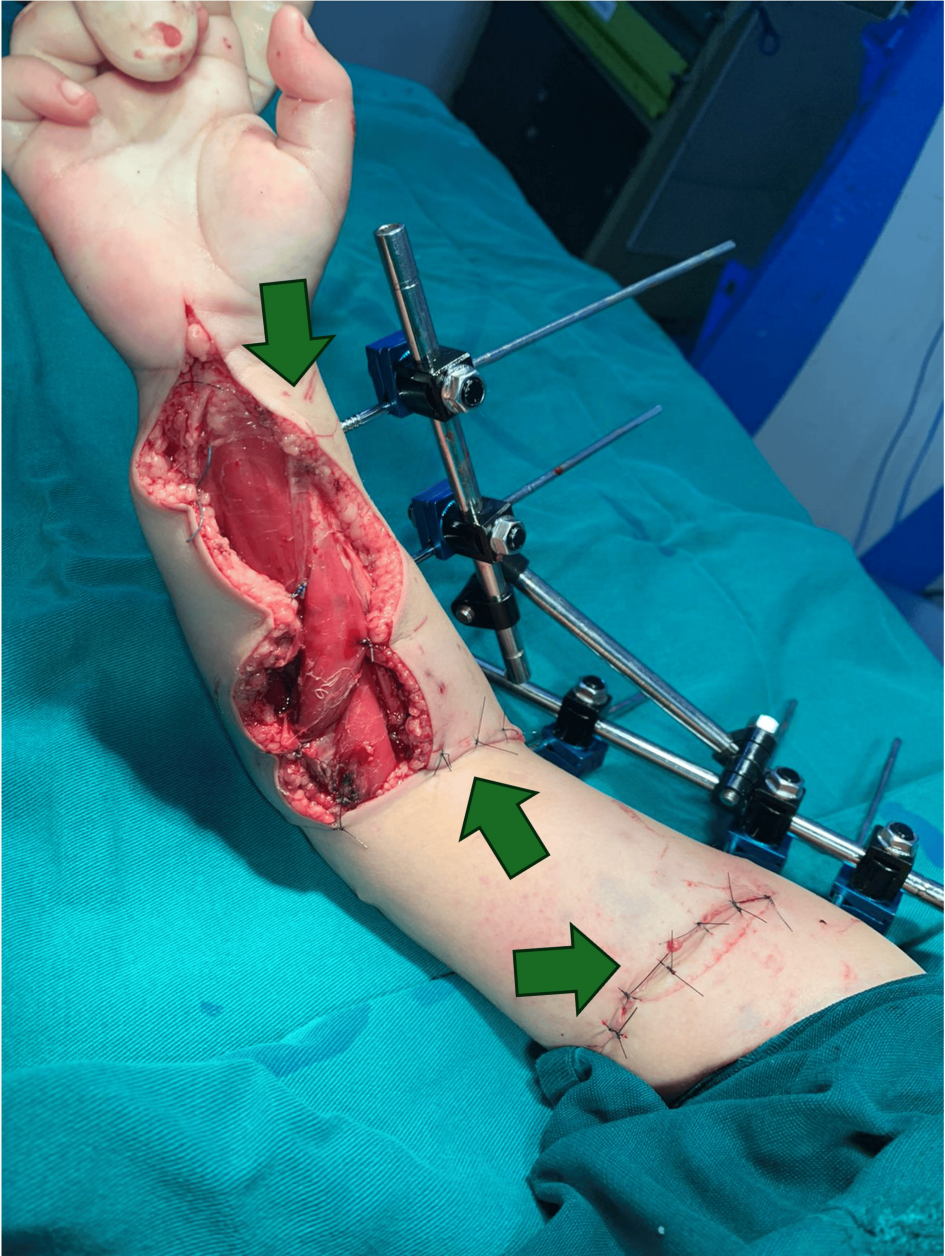
Postoperative optimal soft tissue management after damage control with external fixator stabilization By placing an external fixator correctly, we can support the soft tissue. Cutaneous, subcutaneous, muscular tissue with good perfusion is observed. Coping points on the forearm help to prevent skin retraction.

The patient was promptly admitted to the surgery room for damage control surgery. Under general anesthesia, surgical debridement and lavage of the open fracture were performed, followed by stabilization using a mini external fixator with elbow locking on the day of the trauma. Postoperative X-rays demonstrated an acceptable reduction of the humeral bone injury and alignment of the forearm fractures (Figure 4), with optimal soft tissue management (Figure 5).

**Figure 6:**
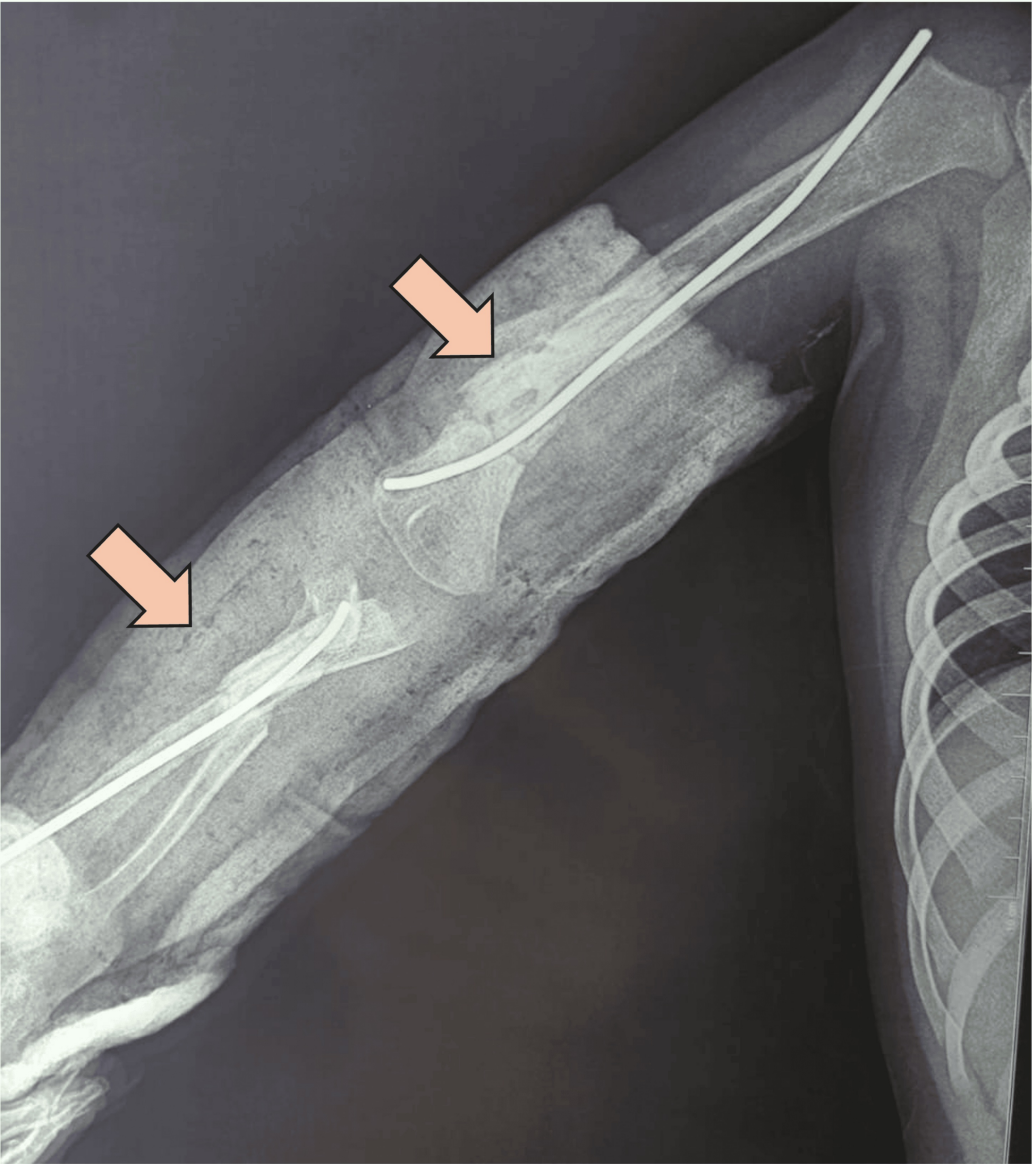
Postoperative X-rays in the anteroposterior (AP) view showing the intramedullary titanium elastic nails (TENs) Acceptable fracture reductions are observed.

**Figure 7:**
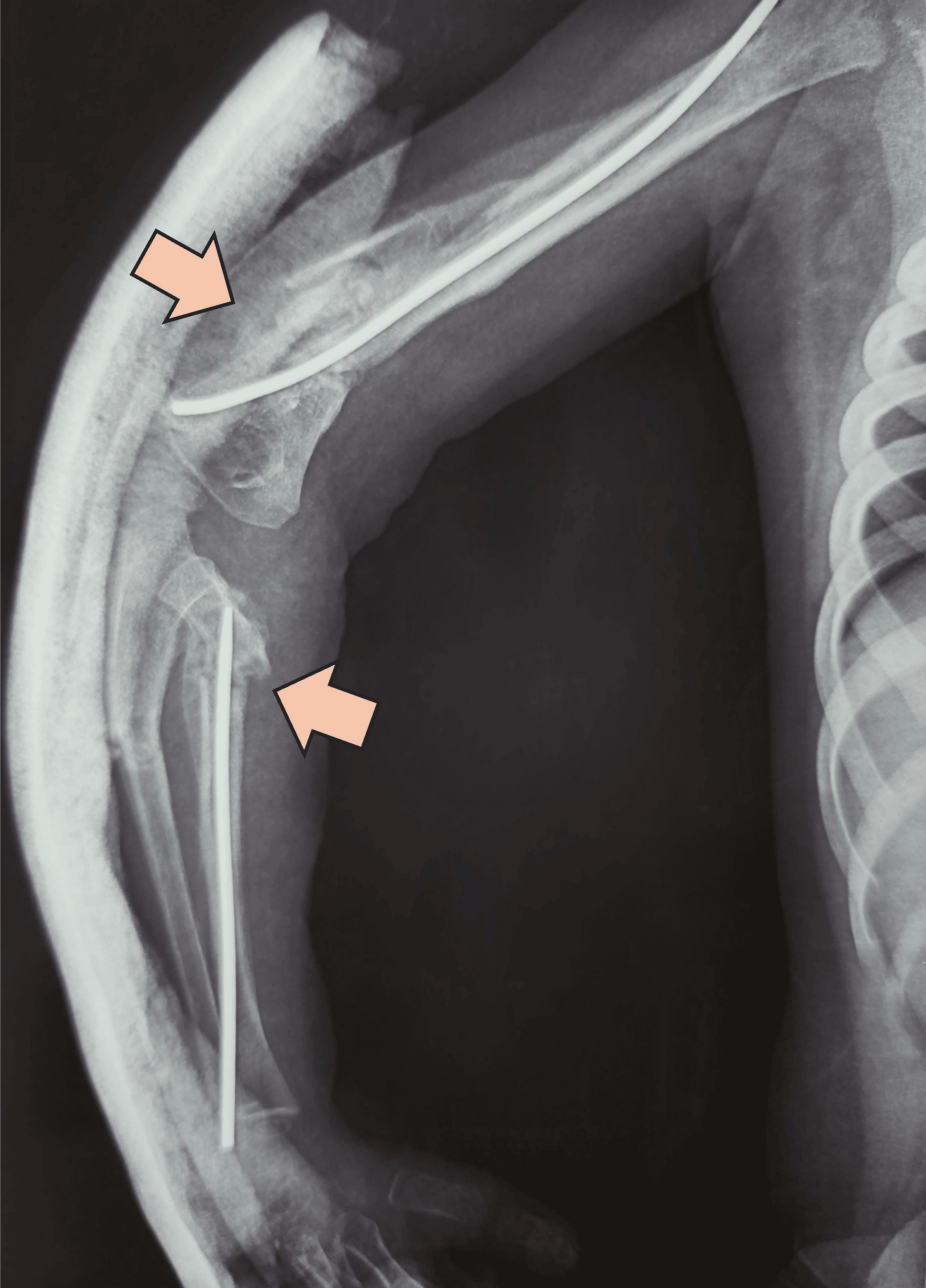
Post-surgical X-rays in the lateral view showing the intramedullary titanium elastic nails (TENs) Acceptable fracture reductions are observed.

**Figure 8:**
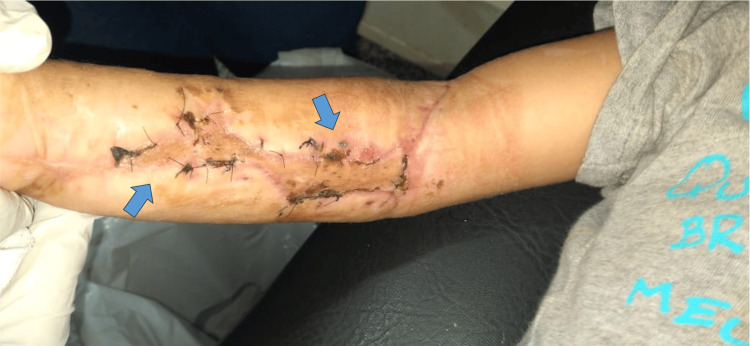
Two-month postoperative follow-up from plastic and reconstructive surgery The graft was found to be in good condition during the outpatient check-up.

Two days post-trauma, the patient underwent a second surgical lavage. Seven days after the incident, the mini external fixator was replaced with fixation using two titanium elastic nails (TENs) through an intramedullary technique due to the fracture trace and the minimal incision required to be introduced, taking into account the state of the soft tissue (Figures 6, 7). Fifteen days post-trauma, the plastic and reconstructive surgery team performed a skin graft for coverage. At the two-month postoperative follow-up, the graft was in good condition (Figure 8).

**Figure 9:**
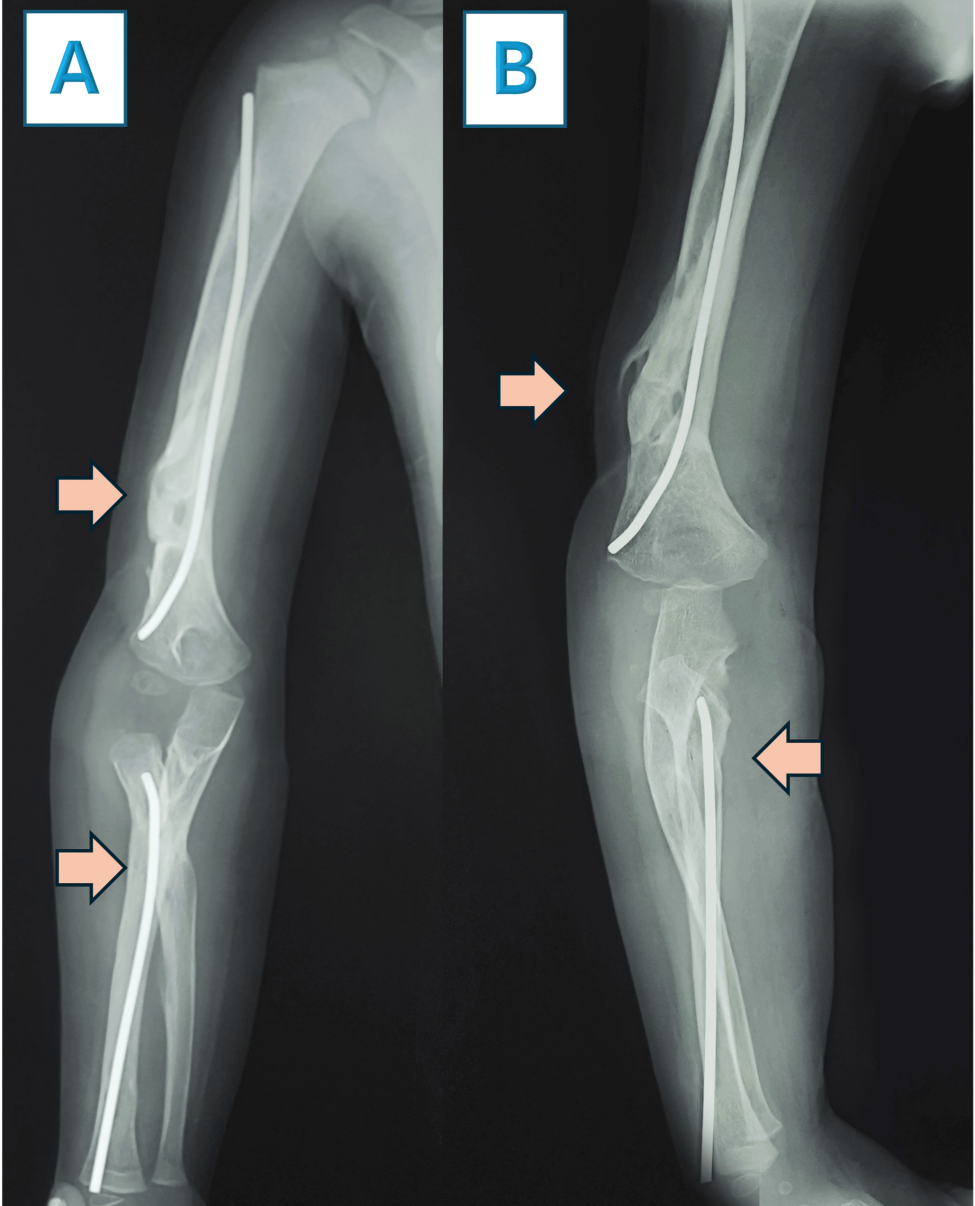
X-rays six months after surgery with titanium elastic nails (TENs), showing in the anteroposterior (AP) and lateral views In image A, there is consolidation from the AP view, and in image B, there is consolidation from the lateral view.

**Figure 10:**
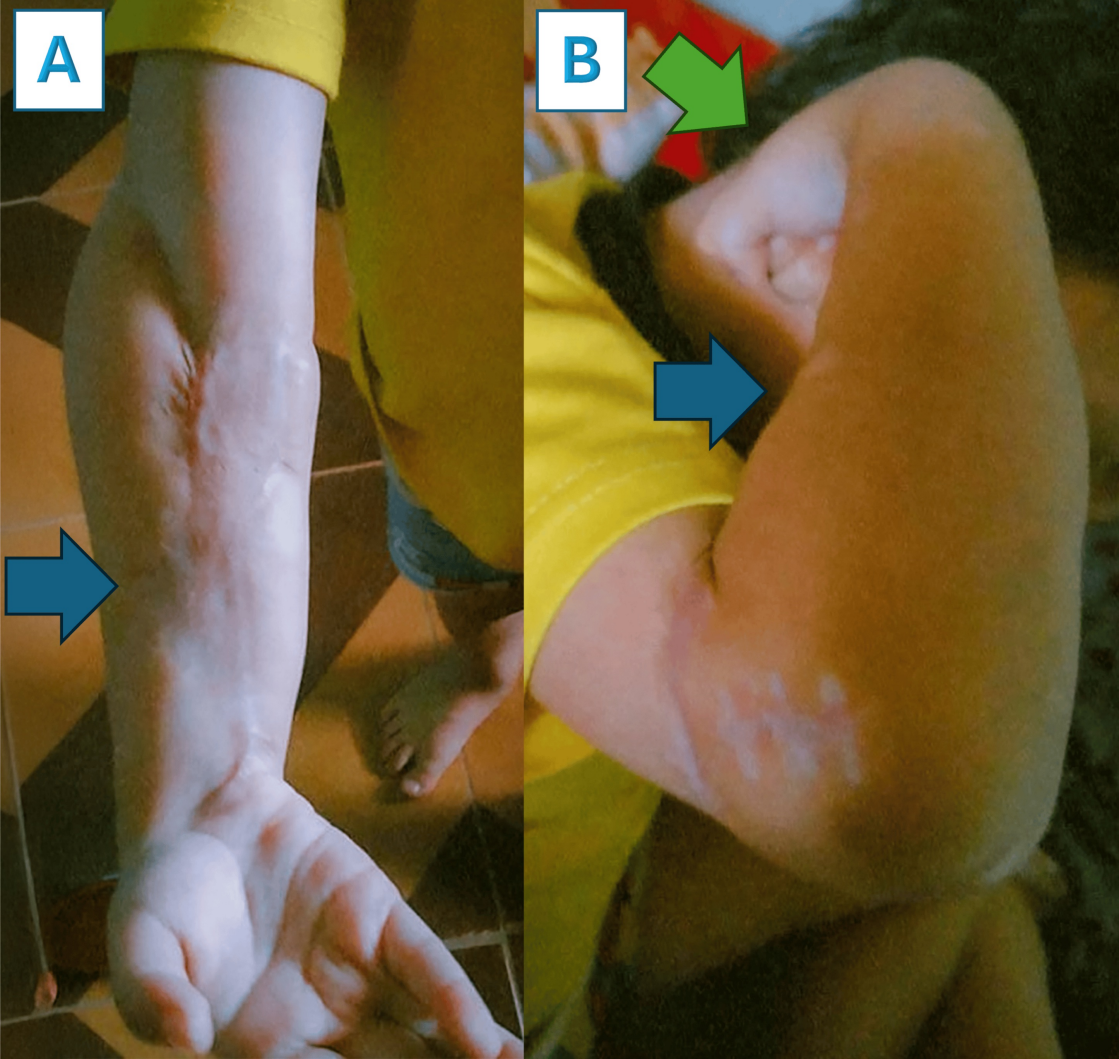
Good range of motion in the elbow has been observed The patient can perform his daily activities. As seen in the image, he can perform full elbow extension (Image A) and flexion (Image B), although there is wrist drop due to radial nerve neuropraxia (Image B, green arrow), with limited pronation and supination.

Six months after the operation, X-rays show signs of fracture healing (Figure 9). The patient exhibited a good range of motion in the elbow, although wrist drop due to radial nerve neuropraxia was noted, with limited pronation and supination (Figure 10). The patient is currently in the recovery phase, not undergoing physical therapy. Since the patient is two years old, the need for a physiotherapeutic rehabilitation process will not make a difference in the evolution, and therefore it was decided to allow recreational activities at home and to monitor the evolution of the injury.

Importantly, there are no disabling nerve injuries, but the neuropraxia persists. The patient is performing daily activities without limitations and is scheduled for TEN removal in the next three months, marking nine months since the trauma.

Currently, the patient is under multidisciplinary management, and in cooperation with the physiatrist, it was determined that the electromyography should be performed nine to 12 months after the initial trauma.

## Discussion

Accidents have been identified as the third leading cause of death globally, as highlighted by the WHO [11]. These incidents are notably more prevalent among males and are particularly frequent in overcrowded households [12]. The advent of smartphones has also been a significant factor contributing to the rise in domestic accidents [13]. The COVID-19 pandemic further exacerbated this issue, with children spending more time at home, leading to an increase in household accidents. The mean incidence of such accidents rose to 18% in 2020, compared to 6% in 2019 [14]. In addition, a study reported that accidental deaths account for 84% of unnatural deaths [15]. In the Netherlands, accidents are the leading cause of non-fatal injuries in children, with a staggering 68% of these incidents occurring in children aged zero to five years. Severe cases have a high mortality rate, ranging from 64% to 72%, with a mean of 68% [16].

Floating elbow, although rare, poses significant challenges due to its high risk of complications, such as increased compartmental pressures that can result in neurological sequelae [17]. Previous reports have documented similar cases, including those involving radial nerve injury or ulnar nerve irritation [18]. However, our case is particularly noteworthy due to its industrial cause and torsional mechanism, which are uncommon in pediatric patients. 

The reason for the radial neuropraxia was caused by the mechanism of the trauma that produced the humerus fracture. In the surgical act, the nerve section was not found and the treatment for the fracture of the humerus did not influence the appearance of neuropraxia. Time is the determining factor in the prognosis for the decrease of neuropraxia [19].

Physiotherapy for radial neuropraxia was not indicated in the pediatric patient due to the fact that recreational activities were insisted on, with supervision of the evolution. Regression of neuropraxia usually occurs with the passage of time [20,21].

The treatment of floating elbow in children remains a topic of debate. One study suggests that conservative treatment may be more beneficial [22], while others advocate for surgical intervention as the superior option [23]. Complications associated with floating elbow, such as radioulnar involvement, have been linked to poorer functional outcomes, underscoring the necessity of surgical treatment and fracture fixation [24].

The improvement of the case 10 months after the injury is demonstrated by the Mayo Elbow Performance Score (MEPS), where it scored 85 points, which is good compared to its score four months after surgery (i.e., 60 points, which was considered regular) [25].

## Conclusions

The "floating elbow" is a rare but significant domestic injury, particularly affecting children, with a higher prevalence in boys and an increase in cases during the pandemic due to greater exposure to household hazards. This injury can lead to nerve damage, such as paralysis, impacting long-term functionality based on the patient's age, the treatment provided, and the quality of follow-up care. In this study, we can demonstrate that the movement of the elbow joint by the good management of the patient is optimal, and it is expected that the child will recover the mobility affected in the wrist by radial neuropraxia.

The study highlights the importance of educating parents about preventive measures to reduce such accidents and improve outcomes for affected children. Early and appropriate management, including immobilization, surgical cleaning, stabilization with external fixators, and early fixation with intramedullary nails, can minimize complications. Future efforts should focus on educational programs and treatment protocols to optimize patient recovery.
